# Creative Futures in Education: Building ‘Imagination Infrastructures’ for Microbiology and Beyond

**DOI:** 10.1111/1751-7915.70284

**Published:** 2025-12-10

**Authors:** Jake M. Robinson, Martin F. Breed, Alexia Barrable, Ariane König, Robin Taylor, Kenneth Timmis

**Affiliations:** ^1^ College of Science and Engineering Flinders University Bedford Park South Australia Australia; ^2^ The Aerobiome Innovation and Research Hub Flinders University Bedford Park South Australia Australia; ^3^ Queen Margaret University, Edinburgh Musselburgh, Scotland UK; ^4^ Department of Social Sciences University of Luxembourg Esch‐sur‐Alzette Luxembourg; ^5^ The Multiple Intelligence School Suva Fiji; ^6^ Institute for Microbiology Technical University Braunschweig Braunschweig Germany

**Keywords:** education, imagination, imagination infrastructure, IMiLI, microbiology

## Abstract

Education is often reduced to the transmission of knowledge, yet in an era of climate disruption, biodiversity decline, and social injustice and unrest, learners require more than facts and skills. They must develop adaptive capacities that enable them to question, critically analyse, imagine, act, and empathise. One such fundamental capacity is *imagination*, which, despite its centrality to scientific discovery, is frequently undervalued in science education, particularly in fields considered ‘hard’ sciences. Microbiology offers a compelling context for better cultivating imagination because its study requires learners to visualise invisible worlds, connect them to ecological and human health, and explore how such knowledge might be applied to societal challenges. Here, we discuss the concept of *imagination infrastructures*—the environments, tools, practices, inner capacities, and symbolic resources that enable collective imagination—as a framework for better embedding imagination into microbiology education and beyond. We illustrate how imagination infrastructures can help democratise learning, expand worldviews, and promote a sense of responsibility, citizenship, and stewardship. Overcoming curricular, cultural, and resource barriers is required. By nurturing imagination as essential infrastructure, education can equip future microbiologists—and citizens more broadly—to navigate uncertainty and co‐create regenerative futures.

## 1 Introduction

Education is key to realising the potential of an individual, but is often viewed simply as the transmission of knowledge. In a world defined by accelerating change—climate disruption, biodiversity decline, pandemics, and geopolitical uncertainty (Lawrence et al. 2024; Rakowski et al. 2025)—education must provide reliable foundations of established knowledge and cultivate the capacities that learners need to question, adapt, and imagine (Nussbaum 2016). Moving beyond memorizing facts, learners must be equipped to navigate uncertainty, think critically, and co‐create sustainable futures (Rieckmann 2017). Indeed, educational theorists have long recognised the limitations of rote learning. Gardner (1983) challenged narrow assessment cultures as ‘westist, testist, and bestist’; Robinson et al. (2021) argued factory‐model pedagogy fails to cultivate necessary creativity; Lave and Wenger (1991) reframed learning as participation in communities of practice. Yet even these progressive frameworks have not adequately foregrounded *imagination* as an essential and adaptive infrastructure for navigating uncertainty.

For microbiology educators, imagination is sometimes treated as peripheral to so‐called ‘hard’ sciences (Kind and Kind 2007). Yet it is imagination that enables learners to conceptualise invisible worlds that play fundamental roles in our ecosystems and health (e.g., the microbiome, or the collection of bacteria, fungi, viruses and others and their ‘theatre of activity’), to ask bold research questions and to envision how science might be applied to societal challenges (Timmis et al. 2025). Without imagination, education remains relatively inert, and with it, it can be a catalyst for discovery, problem‐solving, enhanced reciprocity and action. Despite its centrality to different knowledge systems, imagination is frequently undervalued in science education and under‐nurtured in curricula, leaving a gap between how science should/needs to be done and how it is often taught (Newton and Newton 2010).

Here, we discuss the idea of ‘imagination infrastructures’ (König et al. 2025; Robinson et al. 2025) as a framework for better embedding imagination into education. These infrastructures are networks of practices, tools, spaces, and cultural supports that enable learners and educators alike to think creatively, collaboratively, and regeneratively. Nurturing imagination infrastructures across all levels of education is vital for empowering the next generation to address the interconnected global challenges.

## 2 What Are Imagination Infrastructures?

The concept of imagination infrastructures emerged from interdisciplinary work on the polycrisis—the convergence of climate change, biodiversity loss, inequity, and systemic vulnerabilities. Traditional infrastructures move and mediate material things—water, energy, goods, money, people—across space (Larkin 2013). Imagination infrastructures move and nurture ideas, values, and possibilities (König et al. 2025; Robinson et al. 2025). They encompass the environments, tools, practices, inner capacities, and languages that make collective imagination possible. For microbiology education, this means creating conditions where learners can visualise microbial worlds beyond their senses, connect underappreciated processes to human and planetary health, reframe problems in ways that reveal hidden opportunities and relationalities, and design and test transformative solutions, even at microscopic scales (Nersessian 2010). By better embedding imagination infrastructures into curricula, school facilities, outreach programmes, and professional development, imagination can become an effective educational tool.

## 3 Why Microbiology? The Invisible as a Training Ground for Imagination

**FIGURE 1:**
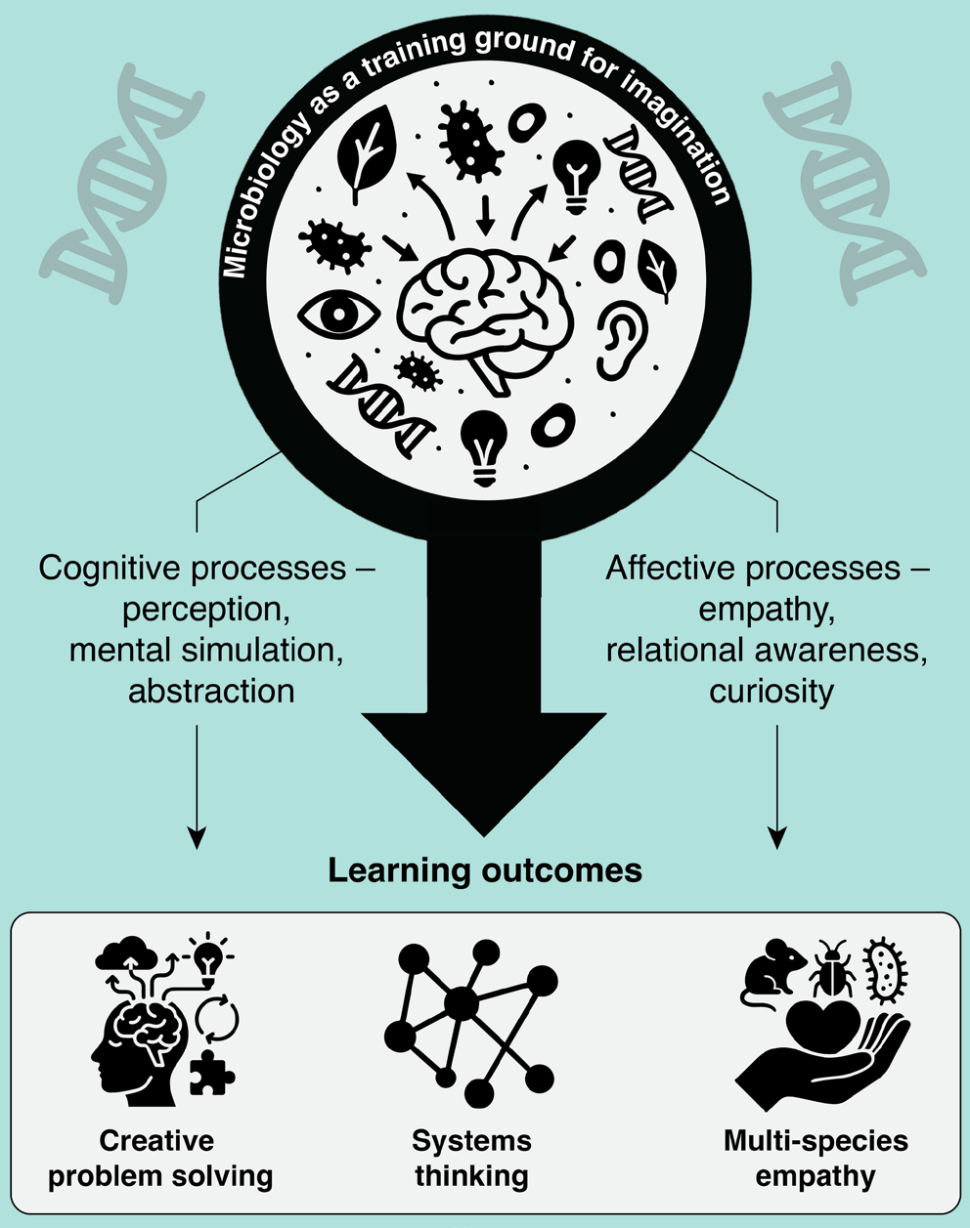
Microbiology as a training ground for imagination.

Microbiology offers a unique context for developing imagination (Figure 1). Unlike many disciplines, the subjects of microbiology are, by definition, invisible to unassisted humans. Therefore, learners must imagine microbial life through models, metaphors, microscopy images, cartoons, and videos, molecular data, and art (Buxeda and Moore 2000; Drew and Triplett 2008; Adkins et al. 2018). This act of imagination is the very basis of scientific reasoning (Meynell 2023). When learners observe gas bubbles breaking on the surface of a stagnant pond, or a colour change in a microbial culture in or on a medium containing a pH indicator, or when they are asked to envision bacteria metabolising carbon in soil, or viruses co‐evolving with their hosts, they are engaging in structured imaginative study.

From a neuroscience perspective, this process draws on the brain's capacity for mental simulation. The same neural networks that activate when we perceive the outside world—the visual cortex, motor areas, and regions of the prefrontal cortex—also light up when we construct mental images of things we cannot see (Pearson et al. 2015; Dijkstra et al. 2019). In effect, learners are ‘seeing with the mind's eye’. Imaginative visualisation recruits overlapping brain circuits with perception and memory, allowing learners to integrate abstract information into vivid mental models (Kosslyn et al. 2001). By exercising these neural pathways, microbiology education can teach scientific content and strengthen the cognitive machinery that supports creativity, as well as problem‐solving, multispecies empathy, and adaptive ‘systems thinking’ in the face of uncertainty.

By explicitly recognising and cultivating this skill, educators can help learners appreciate the creativity inherent in science. Yet, people are afraid of microbes (‘germophobia’) and increasingly shut nature out of their lives (Robinson et al. 2021). This is an unhealthy relationship. Microbiology is central to our lives and to addressing some of the world's most pressing challenges, such as antimicrobial resistance, food security, pandemics, and soil and ocean health (Cavicchioli et al. 2019; Timmis et al. 2019; Ranallo et al. 2021; Robinson et al. 2024; Timmis et al. 2025). Imagination is often required to connect microbial processes to these larger societal systems and to envision healthier ecological views (after all, we are ‘walking ecosystems’, teeming with lifeforms) and new applications—from microbial biotechnology to regenerative agriculture. Moreover, imagination can inspire relational thinking and reframe the human both *within* and *as* an ecosystem, deeply entangled in the web of life and an inseparable part of nature (Robinson et al. 2025). Indeed, deepening our nature connection can motivate us to act in pro‐social and pro‐environmental ways (Duong and Pensini 2023).

## 4 Five Domains of Imagination Infrastructure in Education

**FIGURE 2:**
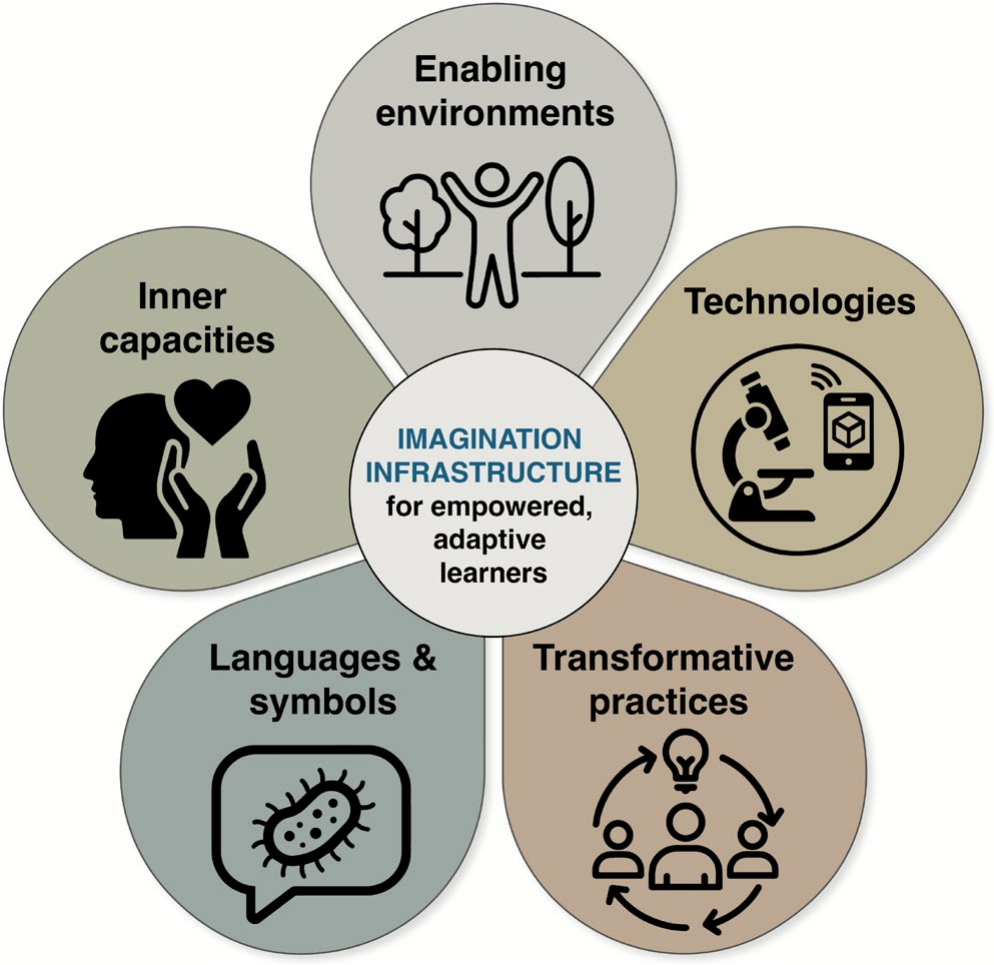
The five domains of imagination infrastructure.

Here, we set out five domains of imagination infrastructure (Figure 2; Table 1) that together illustrate how microbiology education can nurture creativity, and therefore also systems thinking and empathy. (1) Enabling environments such as makerspaces and indoor/outdoor ‘living labs’ encourage creativity and curiosity over rote learning by providing exploratory spaces. (2) Technologies—from microscopes and DNA sequencing tools to augmented reality apps and DIY bio kits—extend perception and make microbial worlds more tangible. (3) Transformative practices engage learners in participatory, problem‐solving activities like hackathons or speculative design challenges. (4) Inner capacities cultivate empathy, resilience, and relational awareness through reflective exercises such as journaling from a microbe's perspective or imagining oneself as a holobiont (which has been shown to increase people's nature connectedness; Robinson et al. 2025). (5) Finally, language and symbols use metaphors, storytelling, and art–science collaborations to reframe how microbes are perceived—as vital partners in life's interconnected systems (whilst acknowledging pathogens). These domains form an integrated framework for imagination‐centred, regenerative education.

**TABLE 1:** Five interconnected domains of imagination infrastructure in education, illustrated through microbiology.

Domain	Description	Examples in education
Enabling environments	Spaces (and excursions) that encourage exploration rather than rote memorisation	Microbiome‐inspired makerspaces where learners design microbial habitats Outdoor ‘living labs’ where learners sample soil/air/water microbes Rotating classroom ‘microbe of the week’ displays with learner‐led curation
Technologies	Tools that extend perception and make microbial worlds tangible. Serious games and virtual realities	Microscopes, DNA sequencing, sonic stimulation tools AR apps showing microbial decomposition in compost or ecosystem processes, or microbes interacting in the gut Low‐cost DIY bio kits for growing and sequencing classroom microbes Smartphone‐based microscopy and citizen‐science apps for microbial tracking
Transformative practices	Iterative, participatory approaches that invite creative problem‐solving, and reframing of prior held beliefs or world views	Speculative ‘future city’ designs integrating microbial restoration and biotechnology for ecosystems, food, waste and health Hackathons where learners design microbial solutions for local sustainability issues
Inner capacities	Practices that cultivate empathy, resilience, relationality and systems awareness	Reflective journaling written from the perspective of a soil microbe Guided meditation imagining oneself as a holobiont—or ‘walking ecosystem’ Reflective group dialogues on microbial interdependence and human health
Language and symbols	Metaphors and narratives that reshape how microbes are perceived	Teaching the microbiome as a community or ‘ecosystem orchestra’ rather than a collection of ‘germs’ Artist‐scientist collaborations and residencies Storytelling workshops that frame many microbes as allies or ‘hidden architects of life’ Comics or graphic novels depicting microbial adventures in ecosystems Music/dance performances embodying microbial rhythms (e.g., fermentation as ‘orchestration’)

## 5 Case Studies

### 5.1 Soil Your Undies and Biodesign Challenge

Citizen science provides a lively demonstration of imagination infrastructures in action. The ‘Soil Your Undies’ challenge (https://cottoninfo.com.au/soilyourundies), where participants bury cotton underwear to visualise soil biota activity, is playful, tactile, and scientifically valid. It democratises microbiology, engages communities, and makes the invisible visible. International education programmes for high school and college learners now introduce the intersection of biotechnology, art, and design and connect them to a global network of leaders in academia, industry, and entrepreneurship to ‘pioneer, envision, create and critique transformational applications in biotech’ (https://www.biodesignchallenge.org/). What role might microbes play in future food systems? What ethical dilemmas could arise? These exercises stretch imagination while grounding it in science. Cross‐cultural learning offers another opportunity. Indigenous knowledge systems can hold deep insights into soils and ecological relationships (Whyte 2013). Ethically integrating these perspectives into microbiology education expands imaginative horizons, demonstrating to learners that science is not culturally neutral but rather enriched by diverse worldviews.

We need to invest effort in designing more engaging citizen science projects that can be carried out globally; this means involving educators from LMICs in the design. Their contextual knowledge is crucial for ensuring that projects are culturally relevant, accessible, and responsive to local priorities, rather than reproducing extractive or top‐down models of science. By involving educators and practitioners from diverse settings early in the design process, citizen science initiatives can better support equitable participation, capacity building, and meaningful data generation that contributes to both global and local sustainability goals.

### 5.2 HUMI‐Suva

A progressive school in Fiji's capital has undertaken a 35‐year longitudinal study to explore the links between environmental microbiomes and human health. Their project is ‘HUMI‐Suva’ (https://humisuva.org). Learners encounter different research phases: collecting soil, extracting DNA, interacting with genomic laboratories, conducting data analysis, and presenting to urban planners. The 7‐year progression develops imaginative capacity developmentally. Year 6 learners collect soil samples, learning about microbial omnipresence through error—initially placing cleaned spades on the ground between samples, contaminating tools without realising it. Year 7 learners extract microbial DNA, learning sterile technique through mistakes like touching their faces with gloved hands. These aren't failures but the process through which learners build mental models of invisible presence. Imagination here is a practical skill: learning to hold in mind the constant presence of what cannot be seen. The longitudinal design teaches thinking beyond individual timescales. Current Year 6 learners will unlikely see the dataset that emerges after decades of collection, yet they participate in creating knowledge that will inform urban planning and health policy long after graduation. This cultivates temporal imagination and contributes to the project, whose outcomes extend beyond direct experience. It also teaches the importance of long‐term endeavours needed to address complex problems and opportunities, the value of contributing to them, and the ‘time’ dimension of systems thinking.

## 6 Overcoming Barriers

Integrating imagination‐focused microbiology education faces systemic obstacles. Curricular constraints reward rote learning over creative exploration—what Gardner (1983) termed “westist, testist, and bestist” education. Robinson and Robinson (2022) argue that education systems must move beyond industrial‐era models to cultivate imaginative capacity. Cultural biases often dismiss imagination as peripheral rather than foundational (Nussbaum 2010). Moreover, resource inequities mean not all schools have access to enabling environments or technologies. To overcome these barriers, educators can integrate imaginative exercises into existing curricula—for instance, lab reports that include creative visualisations—and advocate for assessment systems that reward problem‐solving and creativity. Developing open‐source tools and platforms can also make imagination infrastructure globally accessible, levelling opportunities across contexts.

The *International Microbiology Literacy Initiative* (IMiLI; https://imili.org/) is a good example of how this can be achieved in practice. IMiLI provides open‐access, evidence‐based resources that translate socially relevant microbiological knowledge into engaging, interdisciplinary content for learners at all levels (Timmis et al. 2024). Current efforts to translate the resources into Hindi, Chinese, Spanish and Portuguese will enable their use by more than half of the world's population. By offering freely available teaching materials, story‐driven modules and real‐world case studies, and class exercises that promote consideration of issues such as sustainability, critical thinking in decision making, and stewardship‐citizenship, IMiLI lowers barriers for educators and helps learners connect microbiology to pressing global issues such as climate change, health, food systems and sustainability. Importantly, it demonstrates how imagination infrastructures can be cultivated in ways that are inclusive, scalable and adaptable across cultural and socioeconomic contexts.

Early childhood, a time of rapid cognitive and socioemotional development (Richter et al. 2019), is a great time to introduce imaginative exercises and storytelling. Young children are imagination powerhouses (Kushnir 2022), and we can harness this power of early childhood to embed relational thinking about the world around us, as well as expand on the input of the senses to introduce conceptually correct but age‐appropriate microbiology knowledge and understanding. Picture books and the power of storytelling are allies in creating expanded realities with young children (Roche 2014).

Why does this matter beyond the classroom? Because the world young people are entering is one of profound uncertainty. Climate change, pandemics, and ecosystem degradation are lived realities (REFs). Confronting them effectively requires the full potential of humankind—its understanding of problems, its creativity to develop and implement solutions, and its ability to articulate convincing contextualised reasons to fellow citizens why such solutions are vital. To thrive in such contexts, future citizens need knowledge, but they also need the imaginative capacity to connect dots, reframe problems, envision alternatives, and challenge biased narratives and dogmas. Microbiology, with its invisible domain and global significance, is a fertile training ground. By embedding imagination infrastructures into microbiology education, we can prepare learners to be competent and creative scientists and adaptive thinkers, capable of co‐creating regenerative futures.

## 7 Conclusion

From Pasteur to Margulis, breakthroughs in microbiology have always depended on leaps of imagination. Today, in an age of cascading crises, the stakes are higher. Education must rise to the challenge by treating imagination as infrastructure, that is, essential, intentional, and accessible to all. By cultivating enabling environments, harnessing technologies, embedding transformative practices, nurturing inner capacities, and protecting language, we can turn classrooms into sites of imagination. In doing so, we can empower a generation of learners to navigate complexity, restore ecosystems, and reimagine human–microbial relationships in ways that sustain life on Earth.

Beneath our superficial differences, we are all of us walking communities of bacteria. The world shimmers, a pointillist landscape made of tiny living beings. Lynn Margulis

## Author Contributions

**Jake M. Robinson:** conceptualisation, visualisations, writing – original draft, writing – review and editing. **Martin F. Breed:** writing – original draft, writing – review and editing. **Alexia Barrable:** writing – original draft, writing – review and editing. **Ariane König:** writing – original draft, writing – review and editing. **Robin Taylor:** writing – original draft, writing – review and editing. **Kenneth Timmis:** writing – original draft, writing – review and editing.

## Funding

This research was supported by funding from the Australian Research Council (grant DP250101476), the New Zealand Ministry of Business Innovation and Employment (grant UOWX2101), and the National Environmental Science Program's Resilience Landscape Hub.

## Conflicts of Interest

J.M.R. and K.T. are members of the International Microbiology Literacy Initiative (IMiLI).

## Data Availability

The authors have nothing to report.
